# Obstinate Hemorrhage from Extraction of a Tooth

**Published:** 1878-06-20

**Authors:** S. H. Anderson

**Affiliations:** Mo.


### OBSTINATE HEMORRHAGE FROM EXTRACTION OF A TOOTH.

By S. H. Anderson, M.D., of Mo.

I wish to state the salient points of a case which lately came under my notice, and which, I candidly confess, gave me no little trouble, taxing my medical lore to the utmost.

May 5th, 1878, I was called to see J. L. H——, native of Alabama, aged about 45 years, farmer by occupation, and residing on a creek-bottom where malaria is rife. Was told by messenger that, himself a farmer, had, five days previously, extracted a molar tooth for Mr. H., and that Mr. H. had been bleeding profusely ever since; that is to say, nearly five days. I should have explained that I was called late Saturday evening. On arriving at the house of Mr. H., a truly pitiable and frightful object met my sight. Mr. H., reclining on a bed which was saturated with blood; the floor around his bed being also covered with blood, spat there by himself, while a large night glass at his bedside was filled with blood, this being the third one, I was told by Mrs. H. I found Mr. H. breathing with difficulty through his nostrils, his mouth being filled with clots of blood.

With my fingers I removed the clots from his mouth, which were putrid, and stank horribly. I found that the blood was oozing from every part of his mouth. On inquiry, I learned that Dr. P——, a young man who had, a few months before, graduated, had been in attendance on Mr. H. I was shown a bottle containing oz. vi. of a fluid which I recognized as a tincture of solution of the perchloride of iron. I was told that the doctor had saturated a quantity of cotton batting with the fluid, with which he had filled the mouth of his unfortunate patient, and which, with the assistance of bystanders, he had kept in his patient's mouth by main force, until the wretched man in mortal agony, had cried aloud!

The result of this practice was erosion of the entire mucous membrane of the mouth, and consequent hemorrhage over the entire membrane.

The doctor had left his patient, assuring the family that there was no danger whatever; that Mr. H. might thus bleed a *week* and be the better for his depletion; but to others, after leaving, he reported the case as absolutely hopeless.

By estimate I am satisfied that Mr. H. had lost more than two gallons of blood when first seen by me. I learned, on inquiry, that my patient had nearly bled to death on a former occasion, years ago, and that also one of his brothers came near losing his life from the same cause. Of course I recognized the fact that my patient inherited what is known to authors as the *hemorrhagic diathesis*. Learning that my patient had not slept for several nights, more in consequence, I presume, of fright than any peculiarity of his disease, I prescribed a small dose of morphine, in order to quiet his nervous system and procure sleep. The exhibition of morphine may have been somewhat hazardous in this particular case; yet I deemed it of the first importance for the full treatment of his case, to quiet his overwrought nervous system, by bringing about sleep. I also placed him on the use of ergot in full doses, every three hours. Large blister to nape of his neck and to the temples; synapisms to extremities. As to local treatment, I used different drugs: decoct. kino, tannic and gallic acids, alum solution, acetate lead, etc. I also exibited, from the first, quinine.

The hemorrhage was promptly checked and remained checked for fourteen hours. I had procured slate and pencil on which my patient could write, and thus make known his wants; but being a very passionate man, had transgressed my orders and thus brought on a return of the bleeding.

Believing my patient to be laboring under malarious toximiæ, I had prognosticated a spontaneous cessation of hemorrhage on the *seventh* day, and which had actually occurred.

My patient recovered slowly, having been greatly reduced.

As an after treatment, I put him on the use of iron and quinine, good diet, etc. Hope to hear something from professional brethren as to treatment, pathology, etc., of such cases.

